# Growth and Electrical Characterization of Hybrid Core/Shell InAs/CdSe Nanowires

**DOI:** 10.1021/acsami.3c18267

**Published:** 2024-02-20

**Authors:** Mane Kaladzhian, Nils von den Driesch, Nataliya Demarina, Ivan Povstugar, Erik Zimmermann, Marvin Marco Jansen, Jin Hee Bae, Christoph Krause, Benjamin Bennemann, Detlev Grützmacher, Thomas Schäpers, Alexander Pawlis

**Affiliations:** † Peter Grünberg Institut 9 (PGI 9), Forschungszentrum Jülich, 52425 Jülich, Germany; ‡ JARA-Fundamentals of Future Information Technology (JARA-FIT), 52425 Jülich, Germany; § Peter Grünberg Institut 10 (PGI 10), Forschungszentrum Jülich, 52425 Jülich, Germany; ∥ Peter Grünberg Institut 2 (PGI 2), Forschungszentrum Jülich, 52425 Jülich, Germany; ⊥ Central Institute of Engineering, Electronics and Analytics 3 (ZEA 3), Forschungszentrum Jülich, 52425 Jülich, Germany

**Keywords:** InAs nanowire, III−V/II–VI core/shell NWs, selective-area growth, transport properties, NW-based FET device

## Abstract

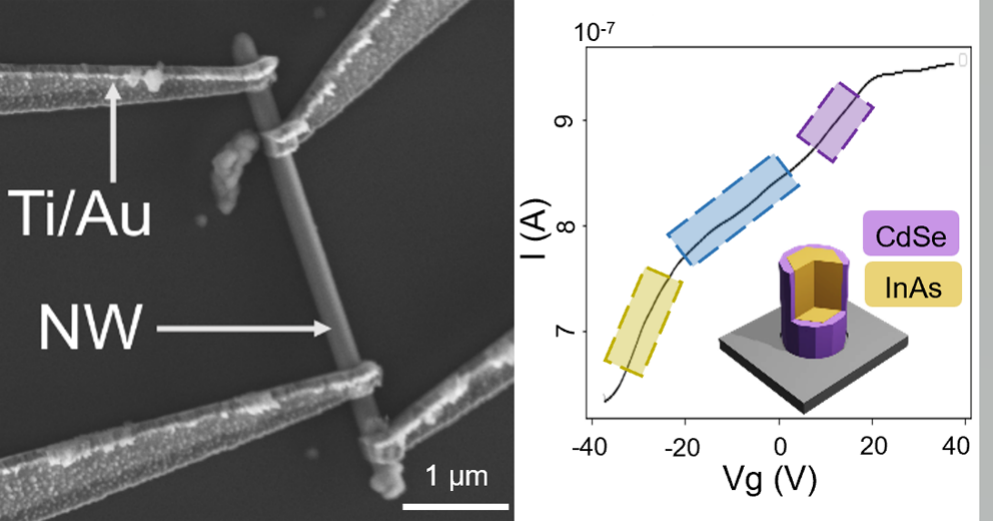

Core-only InAs nanowires (NWs) remain of continuing interest for application in modern optical and electrical devices. In this paper, we utilize the II–VI semiconductor CdSe as a shell for III–V InAs NWs to protect the electron transport channel in the InAs core from surface effects. This unique material configuration offers both a small lattice mismatch between InAs and CdSe and a pronounced electronic confinement in the core with type-I band alignment at the interface between both materials. Under optimized growth conditions, a smooth interface between the core and shell is obtained. Atom probe tomography (APT) measurements confirm substantial diffusion of In into the shell, forming a remote n-type doping of CdSe. Moreover, field-effect transistors (FETs) are fabricated, and the electron transport characteristics in these devices is investigated. Finally, band structure simulations are performed and confirm the presence of an electron transport channel in the InAs core that, at higher gate voltages, extends into the CdSe shell region. These results provide a promising basis toward the application of hybrid III–V/II–VI core/shell nanowires in modern electronics.

## 1 Introduction

The bottom-up approach used for the growth of semiconductor NWs has steadily gained importance in recent years due to the expectation of superior material and surface quality of such nanowires. Especially, if the NWs are formed via a self-assembled growth process, they are not affected by any additional processing treatment during their fabrication.^1−3^ While NWs composed of III–V compound semiconductors have demonstrated various benefits for application in electrical^4,5^ and optical devices,^6−8^ still some sensitive issues remained: The high density of surface states at the nanowire bare surface leads to trapping of charge carriers and hinders control of the charge carrier density with an applied electrical field. Moreover, strong charge carrier scattering at surface imperfections decreases the mobility in the transport channel. One possible approach to avoid the aforementioned drawbacks is the passivation of the core by a suitable shell material grown around the NW core. In recent years, various III–V material combinations have been steadily explored, whereas the shell can provide both, either a larger or a smaller band gap than the core material. Examples for the former case are core/shell GaAs/AlGaAs,^9,10^ InAs/InP,^11^ InAs/AlGaSb,^12^ and the latter case is demonstrated with GaAs/InSb^13^ and GaAs/InAs core/shell NWs.^14^ In both approaches, the transport properties in the NWs are well controllable by engineering the thickness and/or the doping concentration in the shell material.

**Figure 1:**
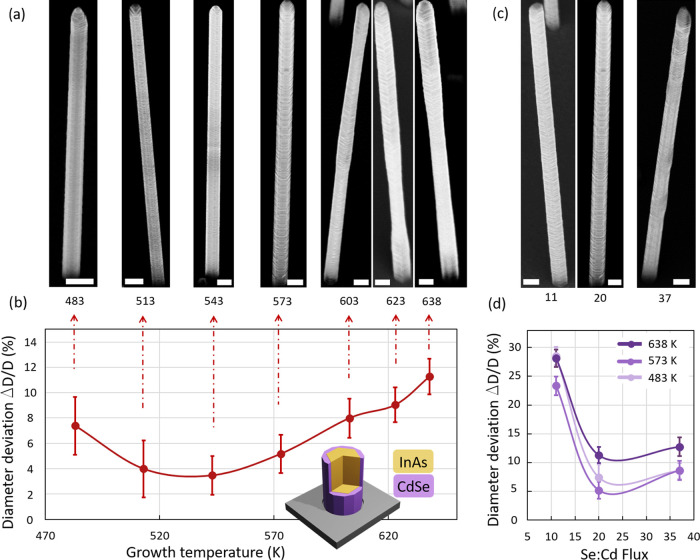
(a) SEM micrographs of NWs as grown at different temperatures in a range between 483 and 638 K and under a constant Se/Cd flux ratio of 20. (b) The coefficient of variation for the NW diameter (i.e., Δ*D*/*D* with Δ*D*—standard deviation and *D*—average of 25 measured points on different NWs) in dependence of growth temperature with the indicated line as a guide to the eye. The inset shows a schematic drawing of the InAs/CdSe core/shell NWs. (c) SEM micrographs of NWs grown under various Se/Cd flux ratios (11, 20, and 37) while keeping a constant growth temperature of 573 K. (d) NW diameter coefficient Δ*D*/*D* as a function of the Se/Cd flux ratio for three different temperatures. All scale bars in this figure correspond to a length of 200 nm. Error bars represent the measurement error of the NW diameter.

In this paper, we report the innovative approach of using CdSe, i.e., II–VI group material, as a shell for the III–V InAs core NW (see inset in Figure 1b). Such a hybrid system containing III–V and II–VI semiconductors is well-known in planar heterostructures^15,16^ but, to our knowledge, has not yet been implemented for NWs. However, in the special case of InAs/CdSe NWs, one first benefits from the small band gap, low electron effective mass, and high g-factor of the InAs core material. Furthermore, the CdSe shell provides a much larger band gap (i.e., 1.9 eV at 0 K) than InAs and additionally leads to a strain-free and atomic sharp interface between the core and shell material due to the extremely small lattice mismatch (approximately 0.5%) in the InAs/CdSe material system. Although the InAs/GaSb system has a similarly low lattice mismatch, its band alignment is type II. Since our InAs/CdSe core/shell heterostructure forms a type-I band alignment, significantly different electrical properties compared to that in the InAs/GaSb system^17^ are expected. Moreover, during NW growth, In atoms diffuse from the InAs core into the CdSe shell and thus provide modulation doping. The doping atoms are remote from the electrons efficiently confined in the core due to the relatively large CdSe/InAs conduction band offset. This enables the control of the core conductivity while avoiding the inclusion of additional scattering centers directly into the conductive channel. Apart from that, the high quality of the CdSe/InAs interface should reduce electron scattering at the interface roughness, which is beneficial for the electron mobility in the core. Taking into account all of the properties of InAs/CdSe described above, it is believed that this system has significant potential for exploring quantum effects and could therefore have future applications in quantum devices.

In the following, the growth and electrical properties of InAs/CdSe nanowires are reported. The first part comprises the epitaxial growth of such NWs and the optimization of the growth parameters. The results of the structural characterization using scanning electron microscopy (SEM) and APT are discussed. In the second part, the fabrication of FET devices from such NWs and corresponding electrical transport measurements are presented. The mobility and carrier concentration were determined, and finally, band structure calculations were performed in order to estimate the nature and spatial localization of the transport channel.

## 2 Experimental Section

The hybrid InAs/CdSe NWs were grown via the self-catalyzed method using molecular beam epitaxy (MBE) on prepatterned silicon dioxide-covered Si-(111) substrates.^18^ First, InAs core-only NWs were grown in a dedicated III–V MBE chamber.^19^ They are free of tapering, the diameter varies in the range of 80 and 200 nm and the length between 2.3 and 5.3 μm, respectively. More details on the InAs NWs are presented in Supporting Information S1. High-resolution transmission electron microscopy (HR-TEM) analysis on individual InAs NWs (not shown here) reveals the presence of segments of both wurzite (WZ) and zinc-blende (ZB) crystal phases. The as-grown InAs core NWs were in situ transferred into a separate II/VI MBE chamber for deposition of the CdSe shell. To achieve a perfect heterogeneous interface between the core and shell and to avoid the potential negative impact of shell inhomogeneities on the electrical properties of the NW, a set of optimal CdSe shell growth parameters was elaborated. Therefore, two series of NW growth were conducted, investigating the influence of growth temperature and material fluxes on the shell quality independently. In the first series, the growth temperature was varied between 483 and 638 K while maintaining a constant Se/Cd material flux ratio of approximately 20. In the second series, the Se/Cd material flux ratio was varied. For all experiments, the flux was measured by a Bayard-Alpert type ionization gauge.

While core InAs NWs show a consistently smooth surface (for details, see Figure S1 in Supporting Information S1), the roughness of the CdSe shell surface considerably depends on the growth temperature of the shell. SEM micrographs of exemplary NWs taken from each growth run are shown in Figure 1a. Decreasing the temperature results in a smoother and qualitatively more homogeneous CdSe shell morphology along the NW. This effect is explained by the presence of both WZ and ZB crystal phase segments of different lengths in the core in combination with a temperature-dependent growth rate of CdSe on those crystallographic phase-dominated regions. At high growth temperatures, a substantial difference in the growth rate of CdSe on the WZ and ZB segments causes the formation of the CdSe shell with varying thickness along the NW. Lowering the growth temperature equalizes the growth rates along the NW, leading to the formation of a CdSe shell with homogeneous thickness.

In order to quantify the optimal shell growth temperature, the coefficient of variation introduced as Δ*D*/*D* with Δ*D* being the standard deviation and *D* the average NW diameter was minimized. For this analysis, five individual core/shell NWs from every growth run were taken, and using the corresponding SEM micrographs, the diameter of each of those was determined at five equidistant positions along the NW. For each growth run, *D* was separately calculated as the mean of all 25 measured diameter values per growth run. The resulting coefficient of variation is plotted for each growth temperature in Figure 1b. The points are interpolated by a cubic spline curve as a guide to the eye, revealing a minimum diameter variation between 503 and 573 K, which corresponds to the optimum range of growth temperatures.

The second NW growth series was performed at three fixed temperatures of 483, 573, and 638 K. For each temperature, the Se/Cd material flux ratio was varied between 11 and 37. SEM micrographs of NWs from the growth runs at 573 K are shown in Figure 1c. Similar to the growth temperature series, the SEM analysis reveals a strong impact of the Se/Cd flux ratio on the inhomogeneity of the CdSe shell for all three growth temperatures. By quantifying the diameter deviation in the same way as previously in Figure 1a,b, the optimal Se/Cd flux ratio was found to be around a value of 20 at a growth temperature of 573 K, as depicted in Figure 1d.

## 3 Results and Discussion

**Figure 2:**
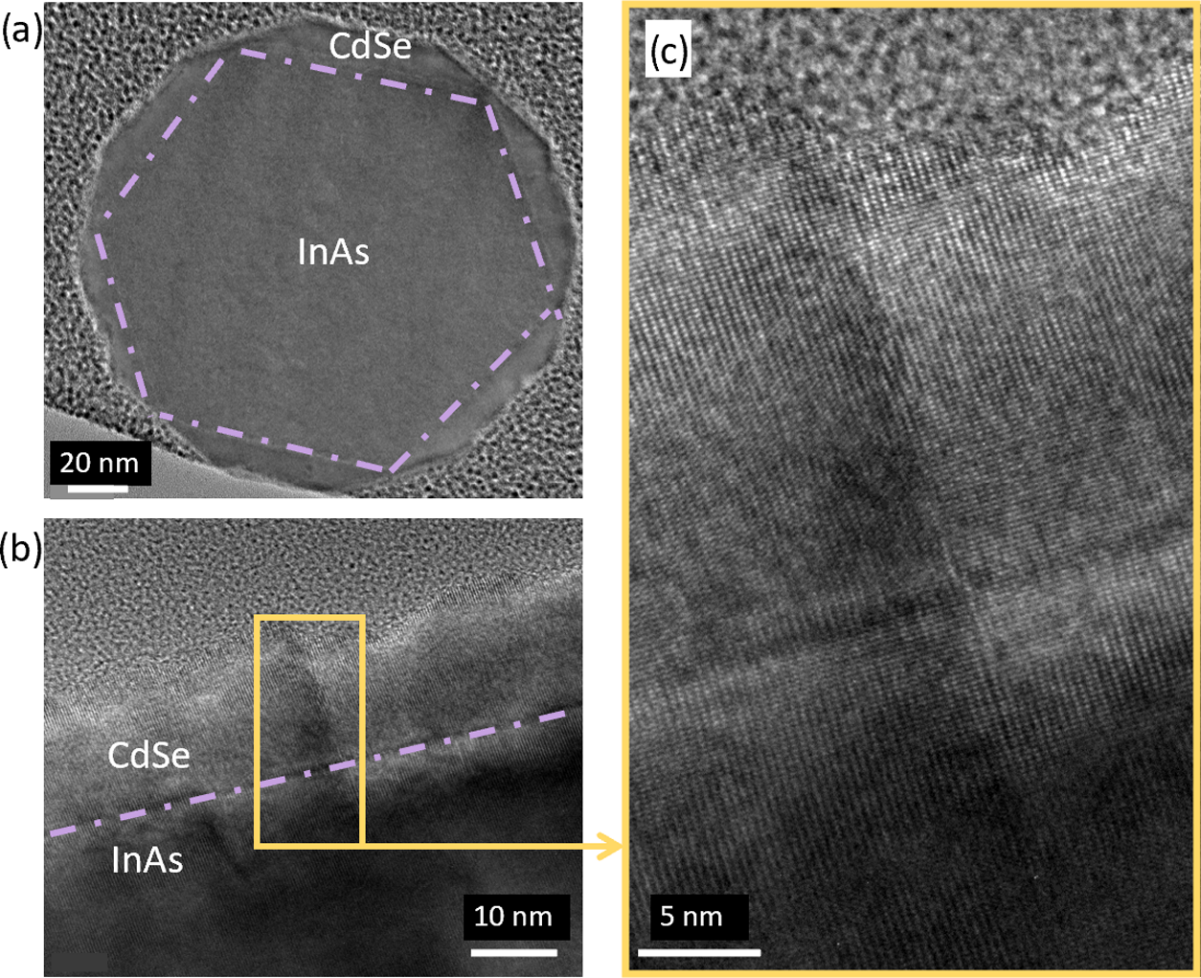
(a) TEM micrograph of an exemplary InAs/CdSe NW scanned across the growth axis in the [111] direction. The dashed purple line indicates the interface between the core and shell. (b) TEM micrograph of the InAs/CdSe interface across the growth axis in the [11̅00] and [112̅] directions for WZ and ZB crystal phases, respectively. (c) High-resolution TEM zoomed-in to the yellow region of (b) in order to highlight the excellent quality of the InAs/CdSe interface region.

For the investigation of both interface quality and crystal structure of the core/shell nanowire, TEM analysis was performed. A cross-sectional TEM micrograph taken in the [111] direction of an exemplary NW grown at 543 K with a Se/Cd flux ratio of 20 is shown in Figure 2a. The image illustrates a hexagonal shape of the InAs core as also reported in ref (20), while the CdSe shell forms a dodecagon with 12 symmetrical facets.^21^

Consequently, it is worth noting here that the effective shell thickness varies along the NW circumference. Figure 2b shows the TEM micrograph across the growth axis in [11̅00] and [112̅] directions for the WZ and ZB phase, respectively, of an exemplary NW grown at 573 K and with a Se/Cd flux ratio of 20. The micrograph confirms the smooth, nearly perfect epitaxial interface between the core and shell. The zoom-in of the interface region reveals the presence of only one crystallographic defect ranging from the core into the shell. Following this observation, we concluded that the shell roughness stems mainly from the different CdSe growth rates on the ZB- and WZ-dominated mixed-phase regions of the core^22^ and is not related to any roughness or imperfection originating from the interface region.

**Figure 3:**
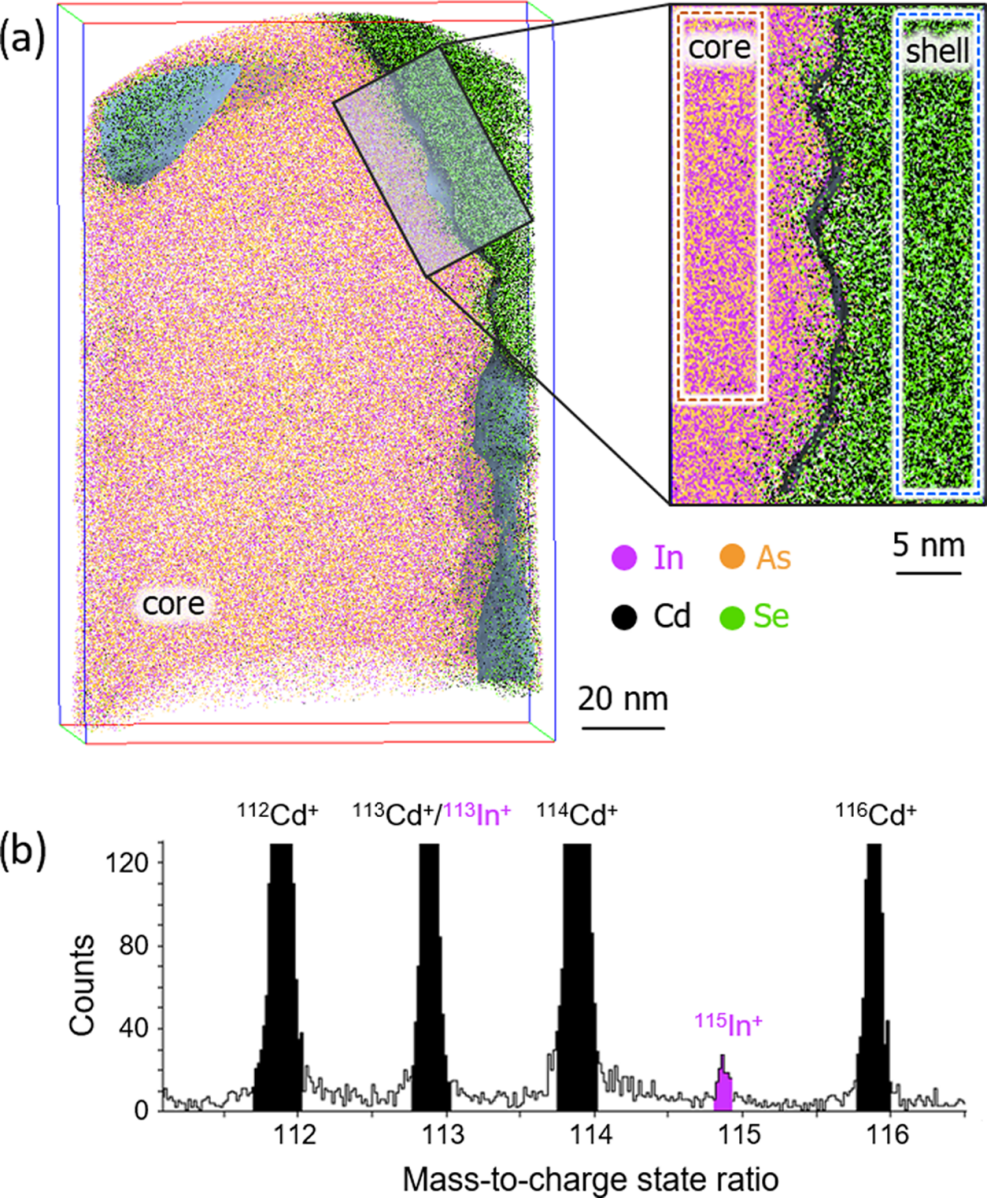
(a) Combined APT elemental map of the NW region containing InAs core and CdSe shell and a magnified inset into the interfacial region. Dashed lines in the inset depict the volumes selected for the compositional analysis. (b) A region of the APT mass spectrum for the shell subvolume depicted by the blue dashed line in (a) demonstrating the presence of In.

Possible interdiffusion of elements between the core and shell was investigated by APT. Figure 3a shows an APT elemental map of a NW region containing the core and a part of the shell, with the inset zooming into the interfacial region and indicating an approximately 5 nm intermixing zone around the interface. It should be noted, however, that the local compositions in the interfacial regions of an APT reconstruction can be distorted due to the local magnification effect.^23^ To exclude the influence of this APT artifact, local compositions in the subvolumes, depicted in the inset in Figure 3a and defined by dashed lines (distant at least 4 nm from the interface), were analyzed. The core subvolume contains no Cd and Se, i.e., no diffusion of the shell elements into the NW core took place. On the contrary, the APT mass spectrum of the shell subvolume shows a clear presence of In (see Figure 3b) with a concentration of 0.12 ± 0.03 atom %, whereas no As was detected in that subvolume. This observation indicates unbalanced In diffusion into the NW shell, which may cause additional n-doping of CdSe. We assume a similar concentration of indium for all NWs in the same growth run since the diffusion effect of indium into the CdSe shell is purely driven by growth time and substrate temperature, which is fixed for a specific growth run. More details about the APT analysis are given in the Supporting Information S3.

In order to perform the electrical characterization of the core/shell NWs, up to four low-ohmic electrical contacts to the NW core were fabricated. First, the NWs were transferred onto highly n-type doped Si substrates by using a micromanipulator tool within the SEM setup. The Si substrates were previously covered by 150 nm of Si oxide in order to form a global backgate to alter the electron concentration in the NW. After the transfer of the NWs, the sample was covered by a thin Si oxide layer via atomic layer deposition (ALD). This layer also serves as a hard mask during subsequent etching steps and prevents the NWs from relocating. Next, holes were etched into the CdSe shell in order to form the ohmic contacts directly on top of the InAs core. Hereby, the SiO_2_ hard mask and CdSe shell were removed via a multistep reactive ion etching (RIE)-based process. At first, contact areas were etched into SiO_2_ using CHF_3_-based RIE chemistry. Afterward, the uncovered part of the CdSe shell was removed via a CH_4_/H_2_-based RIE process. Finally, a short Ar milling step was applied to remove possible residuals and surface oxide. Subsequently, the Ti/Au metal contacts were deposited by a lift-off technique. The process overview with more details along the fabrication process is presented in Supporting Information Section S2.

**Figure 4:**
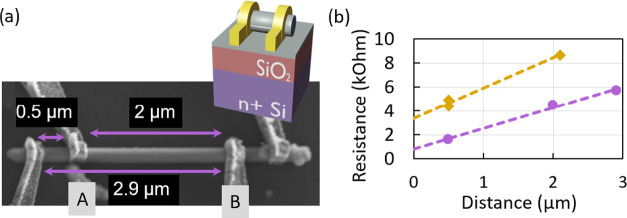
(a) SEM micrograph of a NW after the contact fabrication. Contacts A and B are used for the backgate-dependent measurements presented in Figure 5a. The inset schematically shows the FET device geometry, including the n-doped Si global backgate, which is separated by 150 nm of SiO_2_ from the NW. (b) Distant-dependent resistances for two different NWs. The NWs are taken from the same growth run and were prepared under the same conditions. The highlighted purple dots represent the data from the NW shown in (a) yielding the minimal contact resistance and the dashed purple line is the linear fit between the three obtained data points.

After fabrication of the ohmic contacts, two-terminal resistance measurements on different NWs taken from the same growth run were performed. This resistance comprises contributions from the NW’s conductive channel as well as contacts and wiring resistances. In order to distinguish between these contributions, the current/voltage characteristics was measured as a function of different contact spacings along the individual NWs. Since we are mainly interested in quantum effects, all electrical measurements were performed at 1.3 K. The SEM micrograph in Figure 4a shows an exemplary NW, where three of the four contacts were operable and used for measurements with spacings of 0.5, 2.0, and 2.9 μm.

Figure 4b illustrates the obtained resistances at 1.3 K depending on the contact spacing on different NWs from the same growth run. In order to prevent damage and heating of the nanowire by the bias current, all bias currents were limited to a maximum value of 1 μA. A significant variation in resistances among the investigated NWs is observed (more details are presented in the Supporting Information Section S4). This can be attributed to the presence of a small remaining CdSe barrier below some of the metal contacts due to slight variations in the overall CdSe shell thickness along the NW, so that thicker shell regions have not been completely removed during etching. However, the minimum contact resistance was determined from the NW shown in Figure 4a based on the measurements of three contacts at different distances. The resulting total resistance versus contact spacing is given by the purple dots in Figure 4b and was fitted by a linear function (purple dashed line) in order to extract both the contact and the serial resistance. The intersection of the linear fit with the vertical axis gives a value, which corresponds to the resistance of the two contacts and the cryostat wiring. Taking into account that the resistance of the cryostat wiring is about 160–200 Ω, the obtained contact resistance is in the range of (80 ± 40) Ω. This value is 1 order of magnitude lower than the channel serial resistance of about 4 kΩ. With a NW diameter of 180 nm estimated from a focused ion beam (FIB) cut, a serial resistivity of about 4.2 × 10^–3^ Ω cm was estimated and confirmed that the serial resistance has the dominant contribution to the total measured resistance. The same serial resistivity was also taken to estimate the diameter of another NW, which exhibits a higher contact resistance of around 1.5 kΩ (yellow dashed line in Figure 4b). The calculated diameter of this NW is about 160 nm, which is in good agreement with the total diameter deviation of around 15% estimated from the reference InAs core-only NWs (see also Supporting Information Section S1).

**Figure 5:**
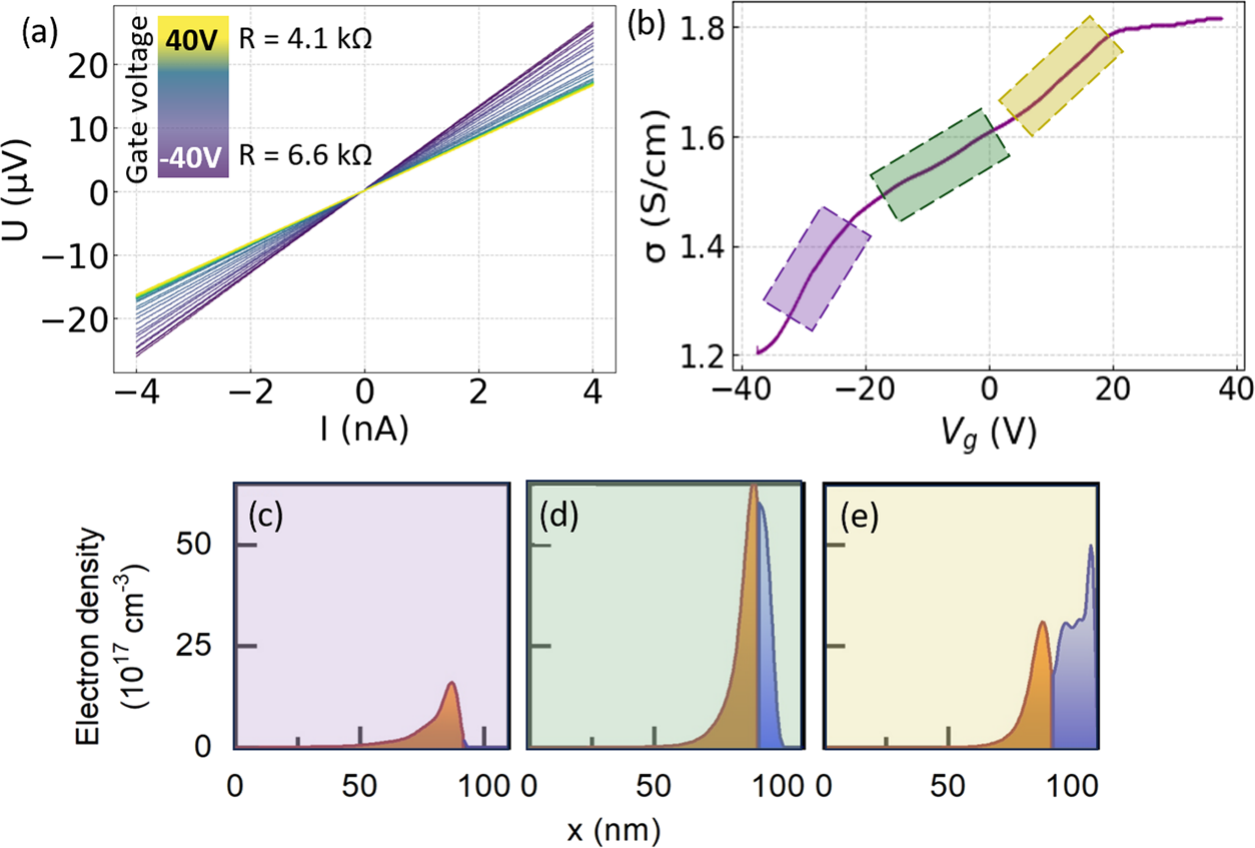
(a) 3-point measured IV curves at 1.3 K taken from the NW shown in (Figure 4a) with backgate voltages between −40 and 40 V. The given resistance values are obtained from a linear fit of the IV curves measured at −40 and 40 V gate voltage. (b) 2-point measurement of the conductivity versus applied backgate voltage at a source–drain voltage of 5 mV. (c–e) Simulations of the electron density for three characteristic regions of the transconductance curve. Colors correspond to the respective areas displayed in diagram (b).

The contacts labeled A and B of the NW shown in Figure 4a were used for measurements of the source/drain voltage as a function of the drain current for different voltages applied to the global backgate (i.e., formed by the Si/SiO_2_ covered carrier substrate). The corresponding dependencies are presented in Figure 5a. The linear characteristics of the measured curves confirm the excellent ohmic performance of the fabricated contacts. The drain current increases with an increase of positive gate voltage, which indicates the n-type conductivity of the NW. The global backgate voltage was tuned in a range between −40 and 40 V, which led to a change of 40% of the measured drain current. However, the complete pinch-off of the conductivity of the NW was not achieved within this applied gate voltage range, which might be partly caused by the considerably high n-type doping concentration in the CdSe shell indicated by the APT measurements.

The electron mobility and concentration within the core/shell NW were determined by measurements of the conductivity at 1.3 K for a constant source–drain voltage of 5 mV while varying the backgate voltage. The obtained transconductance curve is depicted in Figure 5b. It can be divided into three characteristic regions marked in purple, green, and yellow. Every region is described by a different slope of the corresponding linear fit of the *IV* curve and is related to a different transport regime of electrons caused by a specific electron density distribution in the NW.

In order to characterize the origin of the different measured conductivity regions, the electron density in the structure was calculated by solving Schrödinger and Poisson equations for the envelope functions using the effective mass approximation^13,14^ and taking into account the In doping in the shell revealed by the APT analysis. The structural and electronic parameters of CdSe and InAs used for the calculations were taken from references.^24−26^ The conductivity measurements did not show features of the electron ballistic transport in the NW like, for example, the presence of steps on the dependence of the drain current on the backgate voltage. Thus, the electron transport is assumed to have a diffusive character and the conductivity is determined by the product of electron density in the NW core and shell (*n*_core_ and *n*_shell_) and the mobility. Within the relaxation time approximation, the mobility is inversely proportional to the electron effective mass (*m*_core_*and m*_shell_); thus, the total conductivity is proportional to *n*_core_/*m*_core_ + *n*_shell_/*m*_shell_.^13^

The simulation showed that in the purple marked region, nearly all electrons are populating the InAs core (Figure 5c) and the conductance increases rapidly with the applied backgate voltage due to the small electron effective mass in the InAs layer. As a consequence of the remote doping of CdSe with indium, a further increase of the backgate voltage leads to a significant population of the CdSe shell with electrons (green-colored regime), as also evident from the simulations shown in Figure 5d (blue-colored area of the electron density). However, since they have a bigger effective mass in CdSe, the conductance increase is partly suppressed due to the lower electron mobility. For the highest positive backgate voltages (yellow area), the conductance is mainly dominated by the electrons located in the CdSe shell and their effective mass there, as shown in Figure 5e. If the backgate voltage is in the range of 20 and 40 V, the conductance increases only slightly. In that region, the electron density presumably reaches the maximum possible value.

The optimal electron transport regime in the NW is assumed to be realized when electrons populate the InAs core only, which happens for negative gate voltages, i.e., within the purple region in Figure 5b. Then, the electron density can be efficiently controlled by the applied backgate voltage, the electrons are separated from the In dopants in the shell, and due to the high quality of the layer structure, their scattering at the InAs/CdSe interface is negligible. Following the capacitance method described in ref (27), the electron density in this regime was estimated to be around 2 × 10^18^ cm^–3^, which is comparable to that of InAs core-only NWs doped with Si.^28^ In such NWs, the electron concentration scales in the range from mid-10^17^ for the undoped NWs to 3.9 × 10^18^ cm^–3^ for the highest employed Si-doped NWs. The electron mobility of our InAs/CdSe core/shell is approximately 540 cm^2^ V^–1^ s, which is in the same order, but slightly lower than for the core-only InAs/Si NWs, for which mobilities in the range from 780 until 2000 cm^2^ V^–1^ s are reported.^28^ Additional information on the determination of the mobility is provided in Supporting Information S5. The slightly lower mobility is attributed to surface effects, which are likely induced by the 12-facet formation of the CdSe shell, where at the kinks to the InAs core, the CdSe shell thickness of our samples is close to zero. In these regions, we expect a strong interaction of the channel with the surface states, which can lead to the reduced mobility. One possible solution to improve that is to increase shell thickness. Consequently, a substantial increase of the mobility for InAs/CdSe NWs is expected with a larger CdSe shell thickness; however, the detailed investigation of this effect is beyond the scope of this paper.

## 4 Conclusions

In conclusion, the epitaxial growth of self-catalyzed hybrid–III–V/II–VI semiconductor core/shell NWs composed of InAs cores in combination with CdSe shells was successfully established. Although both the core and shell have a mixed-phase crystallographic structure, HR-TEM measurements confirm that a smooth interface between the core and shell was achieved by optimizing the growth temperature and material flux ratios. Moreover, employing APT analysis, the diffusion of In from the core into the shell region was verified and led to a remote n-type In doping in the CdSe shell.

NW-based field-effect transistor devices were fabricated from the as-grown InAs/CdSe NWs to evaluate their transfer characteristics. Investigation of the contact resistances of contacts at different spacings revealed a linear, low-ohmic character with a contact resistance of less than 100 Ω at best. The obtained transfer characteristics indicate the presence of different transport regimes depending on the applied gate voltage. Band structure simulations for different backgate voltages were performed and confirmed the presence of three conductive regimes: By increasing the backgate voltage, the conductive channel relocates from being entirely within the InAs core at negative gate bias, extending into the CdSe shell at zero and moderate positive gate voltages, and finally, saturating and dominating the conductivity for high-positive gate bias.

Overall, the experimental data are in good agreement with the simulations, showing the shift of the electron density from the core to the shell with an increase of the backgate voltage. Therefore, adjusting the backgate voltage allows for tailoring the location of the conductive channel in the remotely doped InAs/CdSe core/shell NWs. Finally, for the case of moderate negative gate voltages (i.e., where the channel is solely located in the InAs core), the electron concentration and mobility were quantified and are in the same order as InAs core-only NWs doped with silicon. Our results may pave the way for hybrid III–V/II–VI core/shell NWs as an attractive material platform, providing flexible and tunable electronic properties having enormous potential for applications in modern electrical devices.

## Supporting Information Available

InAs core-only NW growth; FET device fabrication process; APT experimental setup; mass spectrum analysis of the core; shell and core/shell interface area; electrical characterization of the FET device; mobility calculation (PDF)
