# Supplementary material for: Growth and Electrical Characterization of Hybrid Core/Shell InAs/CdSe Nanowires
Source: ACS Appl Mater Interfaces. 2024 Feb 20;16(8):11035–42. doi: 10.1021/acsami.3c18267 (PMC10910494; doi:10.1021/acsami.3c18267)
Supplement: Supplementary file 1 — am3c18267_si_001.pdf [file am3c18267_si_001.pdf]

Supporting Information

Growth and Electrical Characterization of Hybrid Core/Shell InAs/CdSe Nanowires

Mane Kaladzhian,[*,†,‡] Nils von den Driesch,[¶,‡] Nataliya Demarina,[§] Ivan Povstugar,[‖] Erik Zimmermann[1],[†,‡] Marvin Marco Jansen[2],[†,‡] Jin Hee Bae,[†,‡] Christoph Krause,[¶] Benjamin Bennemann,[¶] Detlev Grützmacher,[†,‡] Thomas Schäpers,[*,†,‡] and Alexander Pawlis[*,†,¶,‡]

†*Peter Grünberg Institut 9 (PGI 9), Forschungszentrum Jülich, 52425 Jülich, Germany*

‡*JARA-Fundamentals of Future Information Technology (JARA-FIT), 52425 Jülich, Germany*

¶*Peter Grünberg Institut 10 (PGI 10), Forschungszentrum Jülich, 52425 Jülich, Germany*

§*Peter Grünberg Institut 2 (PGI 2), Forschungszentrum Jülich, 52425 Jülich, Germany*

‖*Central Institute of Engineering, Electronics and Analytics 3 (ZEA 3), Forschungszentrum Jülich, 52425 Jülich, Germany*

E-mail: m.kaladzhian@fz-juelich.de; th.schaepers@fz-juelich.de; a.pawlis@fz-juelich.de

S1: InAs core-only nanowires

Fig. S1a shows an overview SEM micrograph of typcial InAs core-only nanowires (NWs)[1] grown under the same conditions as the InAs/CdSe NWs. We regularly obtain a yield of 80-90% of NW-filled holes on our fabricated pre-structured substrates. The inset in Fig. S1 is a zoom-in to one of the NWs and reveals its clear hexagonal facets and smooth surface. In order to characterize the geometrical configuration of the InAs NWs we studied SEM micrographs of more than 20 of them and quantified the NW diameter as a function of the NW length. The data is summarized in the diagram in Fig. S1b) yielding a length range of about 3.5 µm–4.8 µm and diameter range of about 115 nm–165 nm, respectively. Note, that the NW diameter is nearly independent from the length, which indicates a large diffusion length of In atoms onto the NW side facets.

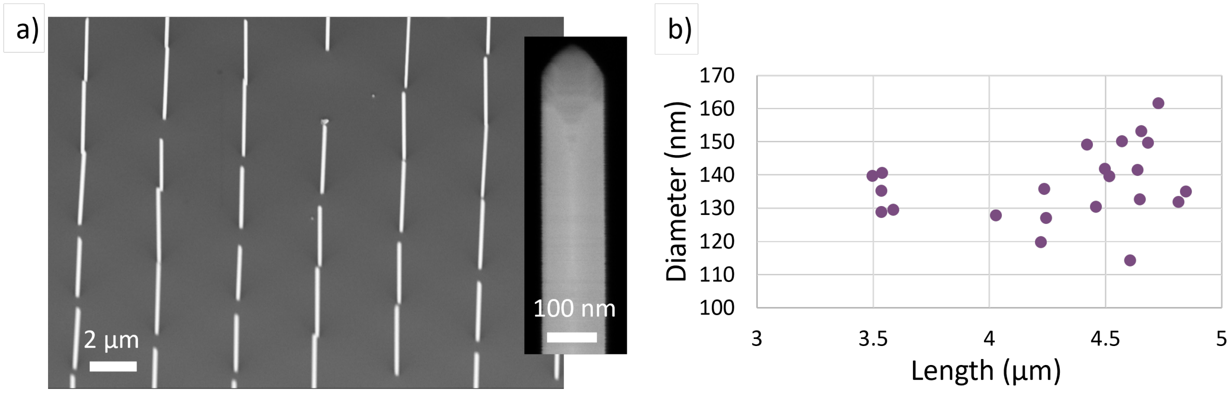

Figure S1: a) Overview SEM micrograph of as-grown InAs core-only NWs. The inset shows the zoom-in to the smooth surface of an exemplary NW. b) Diameter and length evaluation of the InAs core-only NWs taken from the same growth field. (i.e. diameter 80 nm and pitch 4 µm)

S2: Device fabrication

Firstly, the transfer of the NWs to the carrier substrates (n-doped Si-(111) coverered with 150 nm SiO_2) was performed using the micromanipulator installed to our SEM system. This allows for precise postioning and alignment of each NW with respect to the magnetic field.

Subsequent atomic layer deposition of a 30 nm SiO_2 layer on top of the above prepared carrier substrate fixes the NWs at their positions during the subsequent resist coating and etching steps and provides a hard mask for the shell etching.

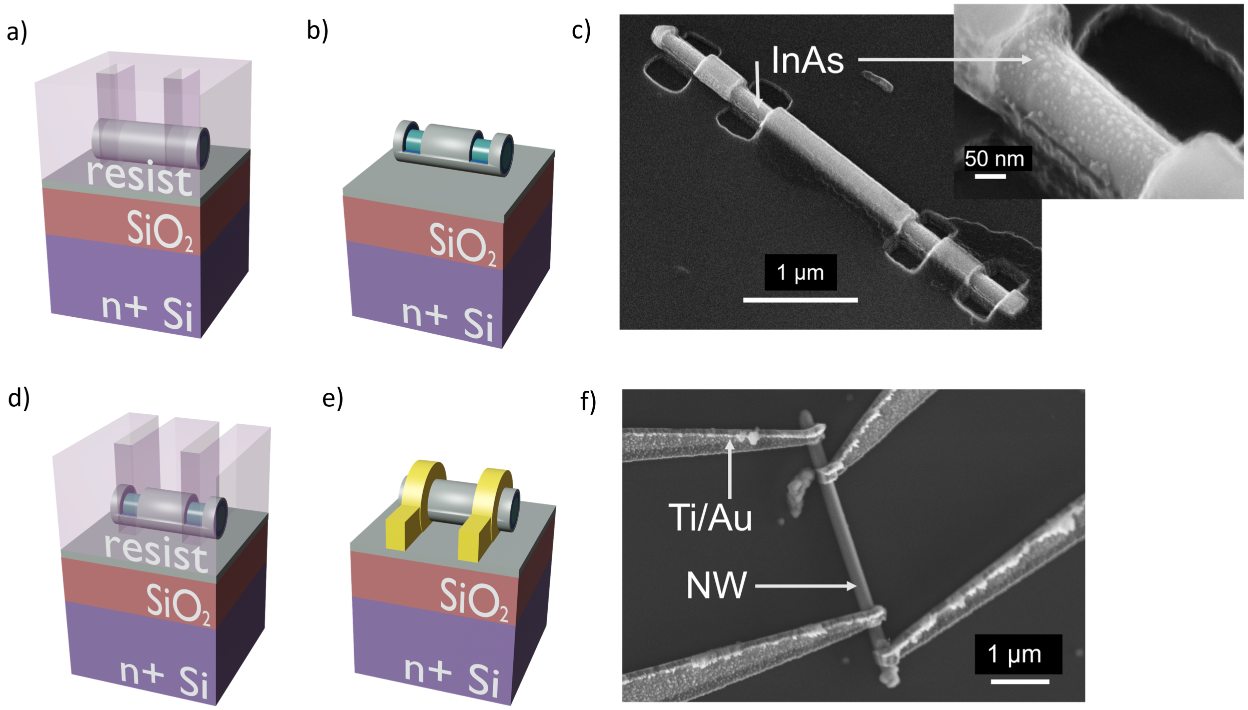

Figure S2: a), b), d), e) Schematic drawing of the fabrication process flow after the transfer of the NWs to the Si/SiO_2 carrier substrate. a) Resist coating to define the contact regions on top of the NW. b) contact openings in the SiO_2 hard mask with bare CdSe shell surface visible. c) SEM micrograph of a NW with the four contact openings etched down to the InAs core (corresponding to the scheme shown in b)). The inset shows a zoom-in to the etched area revealing the InAs surface. d) Second resist coating for the following metallisation of four contacts. e) Formation of Ti/Au ohmic contacts on the InAs core by lift-off technique. f) SEM micrograph of the four metal contacts fabricated by lift-off and directly contacting the InAs core (corresponding to the scheme shown in e))

In order to form up to four local contacts to the two-dimensional electron gas (2-DEG) transport channel at the core/shell interface, CSAR resist was coated at 3500 rpm for 45 sec with pre-acceleration. Subsequent soft-bake was performed at 150°C for 1 min. The CSAR mask was patterned via e-beam lithography (EBL) using a dose of 380 $\mu C\,cm^{-1}$ followed by a cold development for 45 sec at 0°C in AR600-546 solution with subsequent 15 sec dip into IPA (Fig. S2a). The resist mask was utilised to partly etch the SiO_2 layer by a CHF_3-based

reactive ion etching (RIE) with a gas flow of 30 sccm. To avoid the overheating of the resist mask and consequent removal difficulties, the etching was performed in three 30 sec long cycles. After this RIE-process, the resist mask was cleaned in AR-60071 solution followed by 5 min O_2 plasma treatment at 300 W with 200 sccm O_2 gas flow for removing the resist residuals (Fig. S2b). The remaining SiO_2 serves as a hard mask for the subsequent etching of the CdSe shell down to the InAs core by a CH_4:H_2-based RIE process with the gas flows of 10:50 sccm. Figure S2c illustrates an SEM micrograph of the NW after removal of the CdSe shell. The bare InAs surface is visible, the round-shaped features are most likely In-droplets.

As next, metal contacts were defined directly to the InAs core using lift-off technique. At first, a 2-layer stack of CSAR resist was coated, each layer in the same way as for the 1-layer CSAR described above. Then, the resist was patterned via EBL with the 750 $\mu C\,cm^{-1}$ dose followed by the development for 60 sec at 0°C in AR600-546 solution with subsequent 15 sec dip into IPA (Fig. S2d). Afterwards, residuals on the resist mask were removed by a short 10 sec O_2 plasma treatment at 300 W and 200 sccm O_2 gas flow. After 20 sec in-situ Ar-milling, 150/100nm Ti/Au was deposited (Fig. S2e). The final lift-off step was performed in 1:0.3 Dimethyl sulfoxide:Cyclopentanone mixture. The SEM micrograph of the metallised contacts on a NW is shown in Fig. S2f. The successfully fabricated NW-based FET devices were utilized to examine the electrical properties of the InAs/CdSe core-shell NWs described in the paper.

S3: Atom probe tomography

Experimental

APT specimens were prepared in a dual-beam focused-ion-beam (FIB) system (FEI Helios Nanolab 600i). A micromanipulator was attached to an individual nanowire (NW) using electron-beam-assisted Pt deposition, then the NW was broken off from the substrate, transferred to a microtip coupon holder, fixed on the microtip using similar Pt deposition

mode and then the micromanipulator was broken off from the mounted NW. Ga ion beam was not used at any step of the preparation because of possible beam damage. APT analysis was performed using a reflectron-equipped local electrode atom probe tool (LEAP 4000X HR, Cameca Instruments) in laser mode. Laser pulses of 355 nm wavelength, 12 ps pulse length, 1 pJ pulse energy and 125 kHz frequency were applied. The specimen base temperature was kept at 30 K and the ion detection rate was maintained at 0.005 ions per pulse. Data reconstruction and analysis were performed using the Cameca IVAS 3.6.18 software package.

Mass spectrum analysis

Fig. S3 shows the APT mass spectrum of a NW specimen, which includes both core and shell regions. Along with monoatomic ions, multiatomic species of different charge states were detected for Cd, Se and especially As. Table S4 lists the ions for each of the base elements of the NW comprising the entire mass spectrum. It should be noted that identification of certain ions and charge states is impossible due to full peaks overlap (As^+/As_2^{++}, As_2^+/As_4^{++} etc.). Beside the main constituents, minor peaks at 32 and 133 Daltons can be recognized, which seem to correspond to O_2^+ and Cs^+ ions respectively, plausibly originating as processing impurities. These elements are distributed homogeneously over the dataset with the bulk concentrations of <0.03 at.%.

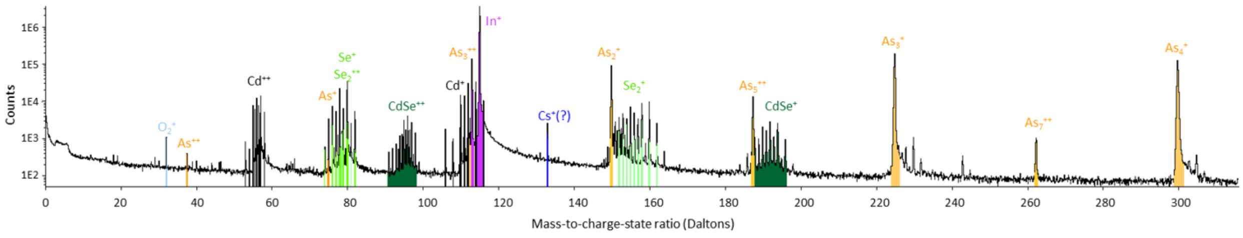

Figure S3: APT mass spectrum of the NW specimen. Both core and shell regions are included.

Element	Ions in the mass spectrum
In	In^+
As	$As^{+/++}$, As_2^+, $As_3^{+/++}$, As_4^+, $As_5^{+/++}$, As_7^{++}, $As^{+/++}$
Cd	$Cd^{+/++}$, $CdSe^{+/++}$, Cd_2Se^{++}
Se	Se^+, $Se_2^{+/++}$, $CdSe^{+/++}$, Cd_2Se^{++}

Figure S4: Ionic species of the NW base elements identified in the APT mass spectrum.

Core-shell interface

Fig. S5 shows proximity histograms (proxigrams) built across the interface, where a compositional transition zone of about 6 nm in width can be observed. Despite the possible influence of the local magnification effect (APT reconstruction artifact) on the core-shell interface, which smears out the true interfacial zone, the measured width appears too large to be caused solely by this artifact, so a compositionally diffuse interface can be assumed from the APT analysis. However, the extent of the artificial interface broadening cannot be reasonably estimated from only APT data without involving other high-resolution techniques, such as HRTEM, so the conclusion about elemental interdiffusion in the near-interface zone cannot be unambiguously drawn from the presented results.

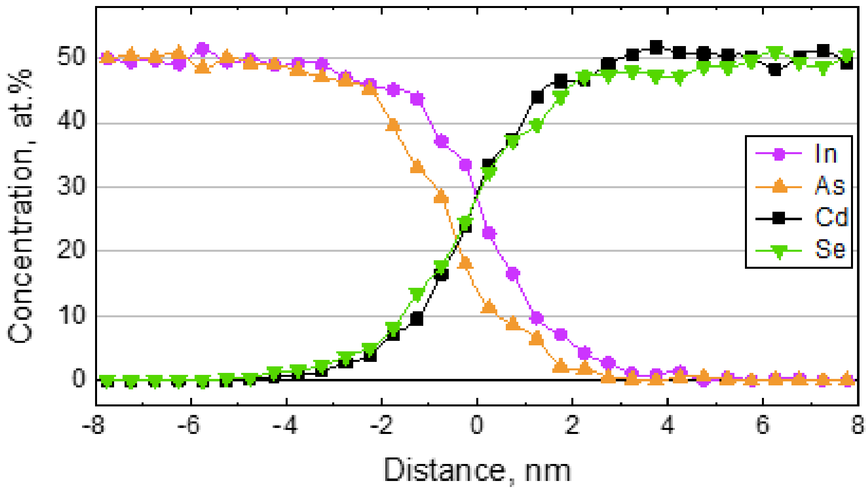

Figure S5: Ionic species of the NW base elements identified in the APT mass spectrum.

It should be noted that the measured elemental concentrations were slightly biased toward the deficiency of As and Se, which is a common issue in APT analysis of III-V or II-VI semiconducting compounds. To account for this bias, the proxigrams shown in Fig. S5 were corrected (normalized) to obtain the correct 50:50 elemental ratios in the bulk of the NW core and shell. Despite this correction, the interfacial region still shows strong excess of In as compared to As, while no deviation from the 50:50 ratio was observed for shell elements (Cd and Sn). This observation aligns with the observed In doping of more distant shell regions, and may indicate an unbalanced electronic state of the interfacial region as well.

Core and shell composition analysis

Fig. S6 shows the mass spectrum of the shell subvolume distant ∼5 nm from the interface (marked with blue dashed line in Fig. 3 in the main paper) in the parts corresponding to the peak positions of arsenic ($As_1 - As_3$) and indium ions. The peak at 150 Da is the only detected peak in this subvolume which could be linked to As-containing ions (As_2^+). However, it overlaps with the peak of a complex Cd_2Se^{++} ion, and the compositional analysis using peak deconvolution procedure evidences no actual As_2^+ contribution into this peak, indicating the overall absence of arsenic in the NW shell. On the contrary, the peak of the main indium isotope (115In) remains clearly present in the shell subvolume, as mentioned in the main text.

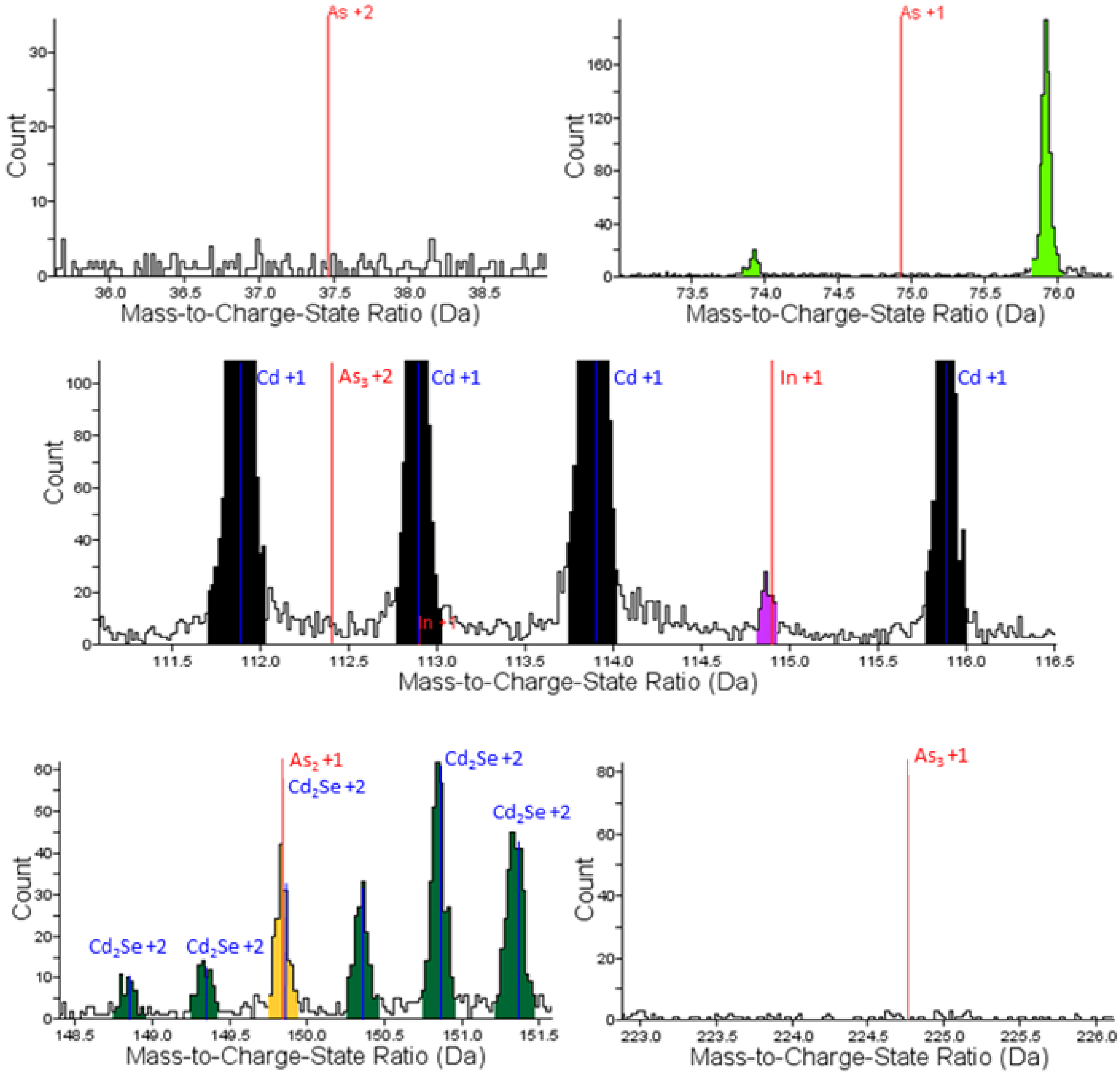

Figure S6: APT mass spectrum - NW shell region.

Fig. S7 shows the mass spectrum of the core subvolume similarly distant ∼5 nm from the interface (yellow dashed line in Fig. 3 in the main paper) in parts which may contain Cd or Se ions. No relevant peaks of these elements were detected, indicating the absence of both Cd and Se in the NW core beyond the interfacial region.

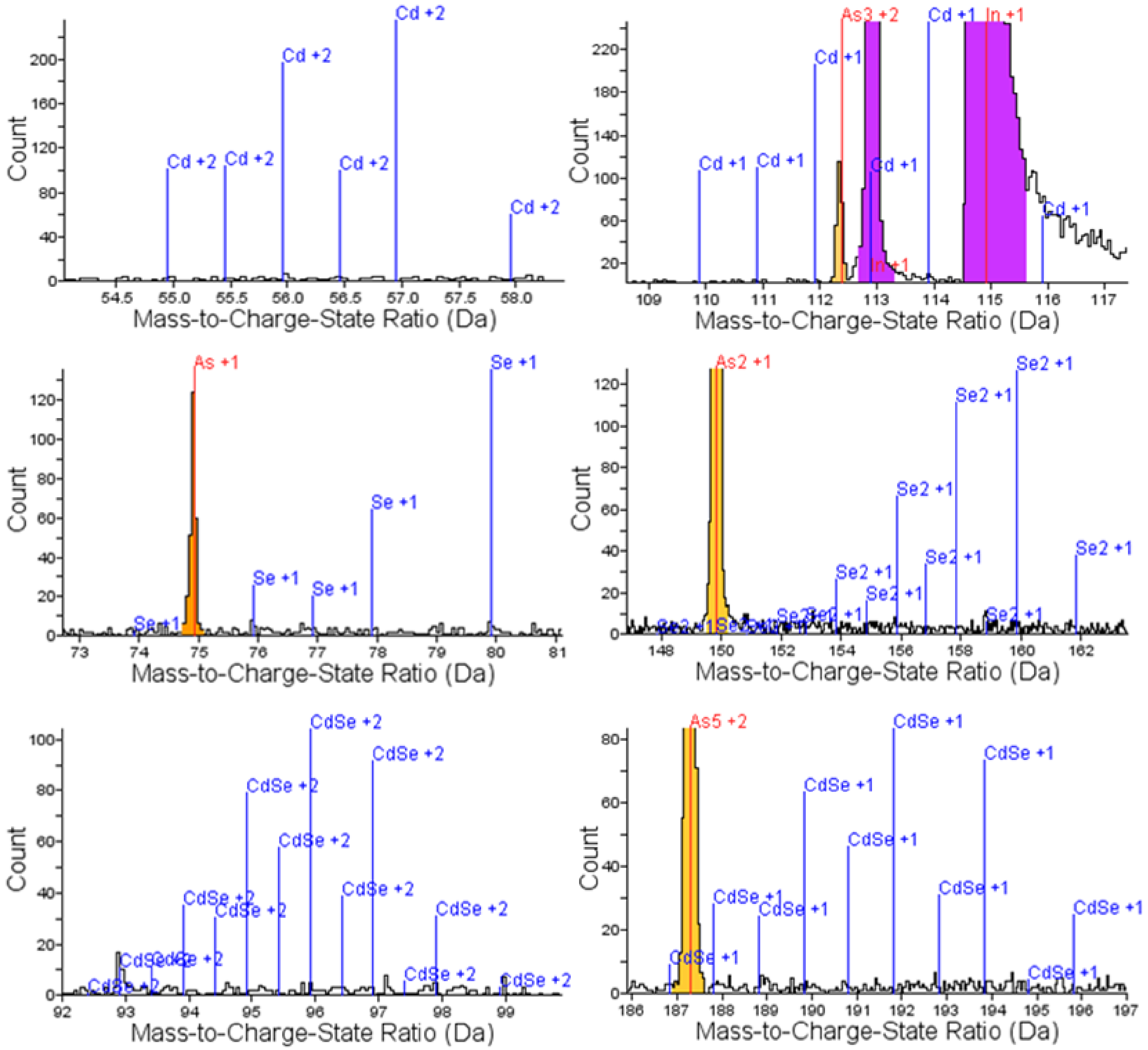

Figure S7: APT mass spectrum - NW core region.

S4: Electrical characterization

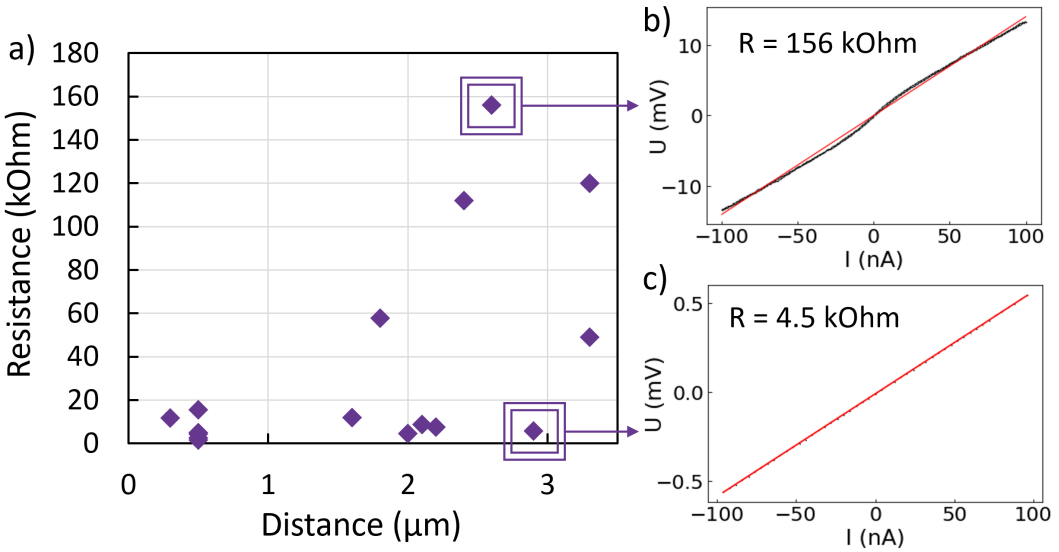

Figure S8: a) Distant-dependent resistances for several different NWs. All NWs are taken from the same growth run and were prepared under the same conditions. b) An exemplary IV curve of 2-point measurement with non-linear behavior. c) An exemplary IV curve of 2-point measurement with ohmic behavior.

Two-terminal current-voltage characteristics were measured for multiple NWs taken from the same growth run (Fig. S8a.). The total resistance for each contact pair was determined by the linear fit of the IV curve and includes the channel resistance, contact resistance and the cryostat wiring. The behavior of the IV curves remains consistent with that in a second cool-down cycle, indicating a resistance variation of 5% only. Despite the same fabrication process, a large range of the measured resistances were observed. This can be attributed to the NW diameter variations (see also section S1) and therefore the different etching results possibly leading to the CdSe barrier formation. A majority of the contacts showed the ohmic behavior. A typical linear IV-curve is shown in Fig. S8c. However some IV-curves are slightly non-linear and yield much higher total resistance, as illustrated in Fig. S8b. The NW with three ohmic contacts was used for the detailed electrical investigation described in the paper.

S5: Mobility calculation

The FET device used in this paper consists of the n-doped Si global backgate separated from the NW by a 150 nm thick SiO_2 dielectric layer. Using such a FET device, the mobility of the conductive channel of the InAs/CdSe core/shell NW is calculated.

According to the calculations in literature[2,3] the mobility of the conductive channel of the NW can be estimated by the following equations. Assuming the NW to be an infinitely long cylinder on a metallic plate, its capacitance C is defined by L - distance between the contacts, ϵ and ε_0 - dielectric constants of vacuum and the gate dielectric, t_{ox} - thickness of the gate dielectric, r - the radius of the NW:

$$C = \frac{2\pi\epsilon_0\epsilon L}{\ln\left[t_{ox}/r + 1 + \sqrt{(t_{ox}/r)^2 + 2t_{ox}/r}\right]}\,. \tag{1}$$

The radius of the NW was measured by the FIB cut after the measurements and was determined to be 180 nm. The distance between the contacts equals 2 µm and was defined during electron-beam lithography masking and additionally confirmed using an SEM scan of the NW.

The mobility of the conductive channel can be defined as a function of the capacitance C, source-drain voltage V_{SD}, L - distance between the contacts and g_m - transconductance:

$$\mu_{FE} = \frac{g_m L^2}{V_{\mathrm{SD}} C}\,. \tag{2}$$

To calculate the transconductance, the source-drain current was measured as a function of the gate voltage in a range from -40 to 40 V with the constant source-drain voltage of 5 mV (Fig. S9). The linearly fitted range corresponds to the conductive regime fully located in the InAs core and therefore is in our main interest. The slope defines the transconductunce value g_m to be equal $10^{-8}\,\Omega^{-1}$.

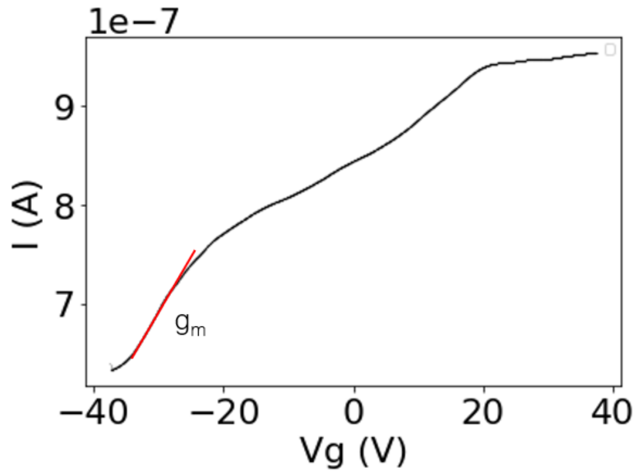

Figure S9: The source-drain current measured as a function of the gate voltage while keeping the source-drain voltage constant and equal to 5 mV. The linearly fitted range corresponds to the regime, where electrical transport is only present in the core of the NW.

Taking into account these formulas, the mobility of the conductive channel in the InAs/CdSe NWs is 540 $cm^2 V^{-1} s$. Discussion on the mobility values is given in the main text of this manuscript.

Present Adresses:

[1] E.Z.: NoKra Optische Prüftechnik und Automation GmbH, Robert-Koch-Straße 6, Baesweiler, Germany

[2] M.M.J.: Eindhoven University of Technology, Groene Loper 19, 5612 AZ Eindhoven, Netherlands

References

(1) Perla, P.; Faustmann, A.; Kölling, S.; Zellekens, P.; Deacon, R.; Fonseka, H. A.; Kölzer, J.; Sato, Y.; Sanchez, A. M.; Moutanabbir, O.; Ishibashi, K.; Grützmacher, D.; Lepsa, M. I.; Schäpers, T. Te-doped Selective-Area Grown InAs Nanowires for Superconducting Hybrid Devices. *Physical Review Materials* **2022**, *6*.

(2) Wunnicke, O. Gate Capacitance of Back-Gated Nanowire Field-Effect Transistors. *Applied Physics Letters* **2006**, *89*, 083102.

(3) Dayeh, S. A.; Soci, C.; Yu, P. K. L.; Yu, E. T.; Wang, D. Influence of Surface States on the Extraction of Transport Parameters from InAs Nanowire Field Effect Transistors. *Applied Physics Letters* **2007**, *90*, 162112.
